# A scoping review of implementation considerations for harm reduction vending machines

**DOI:** 10.1186/s12954-023-00765-2

**Published:** 2023-03-16

**Authors:** Erin Russell, Jessica Johnson, Zach Kosinski, Callie Kaplan, Nicole Barnes, Sean Allen, Emily Haroz

**Affiliations:** Baltimore, USA

**Keywords:** Harm reduction, Naloxone, Overdose prevention, Implementation science, HIV prevention

## Abstract

**Background:**

Community-based harm reduction vending machines (HRVM) are not new to the field of public health; numerous countries have implemented them in response to the needs of people who use drugs over the last three decades. However, until recently, few existed in the United States. Given the rapidity with which communities are standing up harm reduction vending machines, there is a pressing need for a consolidated examination of implementation evidence. This scoping review summarizes existing literature using multiple implementation science frameworks.

**Methods:**

The scoping review was conducted in five stages including (1) Identify the research question; (2) Identify relevant studies; (3) Select the publications based on inclusion/exclusion criteria; (4) Review and extract data; and, (5) Summarize results. PubMed, Embase, and Web of Science were searched and authors screened publications in English from any year. Data were extracted by applying implementation constructs from RE-AIM and the Consolidated Framework for Implementation Research (CFIR). Both frameworks provided a useful lens through which to develop knowledge about the facilitators and barriers to HRVM implementation. The review is reported according to PRISMA guidelines.

**Results:**

After applying the full inclusion and exclusion criteria, including the intervention of interest (“vending machines”) and population of interest (“people who use drugs”), a total of 22 studies were included in the scoping review. None of the studies reported on race, making it difficult to retroactively apply a racial equity lens. Among those articles that examined effectiveness, the outcomes were mixed between clear effectiveness and inconclusive results. Evidence emerged, however, to address all CFIR constructs, and positive outcomes were observed from HRVM’s after-hour availability and increased program reach.

**Recommendations:**

HRVM implementation best practices include maximizing accessibility up to 24 h, 7 days a week, offering syringe disposal options, ensuring capability of data collection, and allowing for anonymity of use. Organizations that implement HRVM should establish strong feedback loops between them, their program participants, and the broader community upfront. Considerations for future research include rigorous study designs to evaluate effectiveness outcomes (e.g. reduced drug overdose deaths) and examination of HRVM reach among ethnic and racial communities.

## Background

With the United States (US) overdose crisis worsening amid a sustained COVID-19 pandemic, particularly among Black and American Indian/Alaska Native communities, harm reduction organizations are urged more than ever to creatively meet the needs of people who use drugs (PWUD)^1^ [1–4]. Implementing harm reduction vending machines (HRVM) stocked with harm reduction supplies is one strategy to increase supply access and reduce syringe sharing [5, 6]. HRVMs complement, and do not duplicate, existing points of supply access thereby increasing the reach of programs among PWUD [7].

HRVMs are similar to drink or snack vending machines and are used in clinical settings to monitor dispensing of controlled substances. Denmark opened the first community-based HRVM in 1987, quickly followed by Norway’s launch of a pilot program that same year [8]. There are now hundreds of HRVM stationed in at least seven countries in Western Europe: Cyprus, Denmark, France, Germany, Luxembourg, Switzerland, and the United Kingdom [9]. The first HRVM in the US emerged in 2009 in Puerto Rico [10], a US territory, and eventually a more comprehensive program developed in Nevada, in the southwestern continental US, in 2017 [11].

Since the onset of the COVID-19 pandemic in 2020, HRVMs have expanded in the US as a way to distribute harm reduction supplies in a contactless manner [12, 13]. Contemporary HRVMs are located in public or semi-public settings and typically managed by a public health agency or a community-based organization. To access supplies, a registered participant of the community-based organization or, in some cases, members of the public, approach the machine and select the desired type and number of supplies. Machines can be programmed to track the items obtained by an individual through use of unique codes, and by extension, support analyses of supply dispensation.

Understanding HRVM implementation is increasingly important as more US communities adopt them. Implementation Science frameworks develop knowledge of how HRVM were implemented and elucidate key lessons that can guide contemporary efforts. To date, there has been one literature review of HRVM, and it did not apply an implementation science lens [14]. Authors identified RE-AIM and the Consolidated Framework for Implementation Research (CFIR) as the most relevant Implementation Science frameworks to guide the review. In recognition of growing racial and ethnic disparities in overdose deaths, and to assess equity considerations during implementation, this review additionally aimed to apply a racial equity lens [15]. Ultimately, this review informs contemporary HRVM programs by deepening understanding of implementation considerations.

## Methods

### Search strategy

The scoping review took five steps: identify the research question, search for relevant publications, screen them, review and chart outcomes, then summarize and report results. The authors applied Preferred Reporting Items for Systematic Reviews and Meta-Analyses (PRISMA-ScR) guidelines and consulted with a research librarian to ensure a comprehensive search strategy. Authors searched PubMed, Embase, and Web of Science, limited it to articles published in English (or that had an available translations), and with no time restraint; the range of dates included 1980–September 2022. This review examines published peer-reviewed literature, not conference abstracts, newspaper articles, nor reports by public health agencies.

After initially searching broad terms such as “harm reduction”, “syringe” or “needle” and “vending machine,” authors collaboratively refined the search terms with controlled vocabulary (e.g., adding in “automatic syringe dispensing machine” to account for common words in Australia), MeSH (medical subject headings) terms, and other keywords. The authors also searched reference records from articles to identify additional references for review; however, that did not yield additional results.

### Inclusion and exclusion criteria

Authors included vending machines used for harm reduction purposes, any vending machine project where the target was PWUD, PWID, staff that plan to or already manage an HRVM, or the community in which an HRVM was placed. Publications were excluded if they did not include relevant terms in the title or abstract, and then the screening criteria was applied to the full text. Authors excluded publications from studies based in a clinical setting and those that evaluated the access of food, snack, drinks, or tobacco through a vending machine. Further, publications that evaluated the overall effectiveness of harm reduction programs but did not focus exclusively on vending machines were excluded.

### Screening procedure

Two authors conducted independent title and abstract screenings of all 85 retrieved titles. Raters consulted one another and in some instances, the full study team, to reconcile differences and build consensus on the selected eligible publications. The same two raters then conducted full text screening on 22 remaining publications.

### Implementation science frameworks: CFIR and RE-AIM

This scoping review applied CFIR and RE-AIM, well-established Implementation Science tools. Using both frameworks provided a useful lens through which to identify lessons learned from decades of implementation abroad; moreover, they provide structure for the translation of historical experience to contemporary application. Use of these frameworks was strategic to create recommendations for US programs interested in HRVMs as a response to the overdose crisis and COVID-19 pandemic.

#### Consolidated framework for implementation research (CFIR)

CFIR provides a practice guide for understanding the myriad factors contributing to the success of public health programs. Those factors that facilitate or hinder a program are organized across five domains, called Intervention Characteristics, Inner Setting, Outer Setting, Individual Characteristics, and Process. When used to its full potential, the CFIR lends support to the development of unique, context-specific logic models for a program’s implementation [16].

#### RE-AIM

The RE-AIM framework addresses limitations of CFIR by including domains that affect the individual intended to benefit from a public health program. Reach, Effectiveness, and Maintenance operate at the individual-level, and Adoption, Implementation, and Maintenance focus on the staff and organization levels [17, 18].

### Data extraction

The research team developed a Microsoft Excel chart to collect extracted data over two phases. The first involved documentation of year and country of publication, sample size, population, and methods of data collection, alongside a RE-AIM content analysis in which reviewers noted if articles included mention of populations reached, a racial equity lens, or a measurement of effectiveness.

In phase two, the authors applied CFIR using a rapid framework analysis modeled after the categorizing methodology described in Nevedal et al. [16]. Authors identified and extracted quotes related to as many CFIR constructs as possible and entered them into the chart. At least two authors read each article, with one conducting initial extraction and the second verifying the findings.

## Results

### Yield

**Fig. 1:**
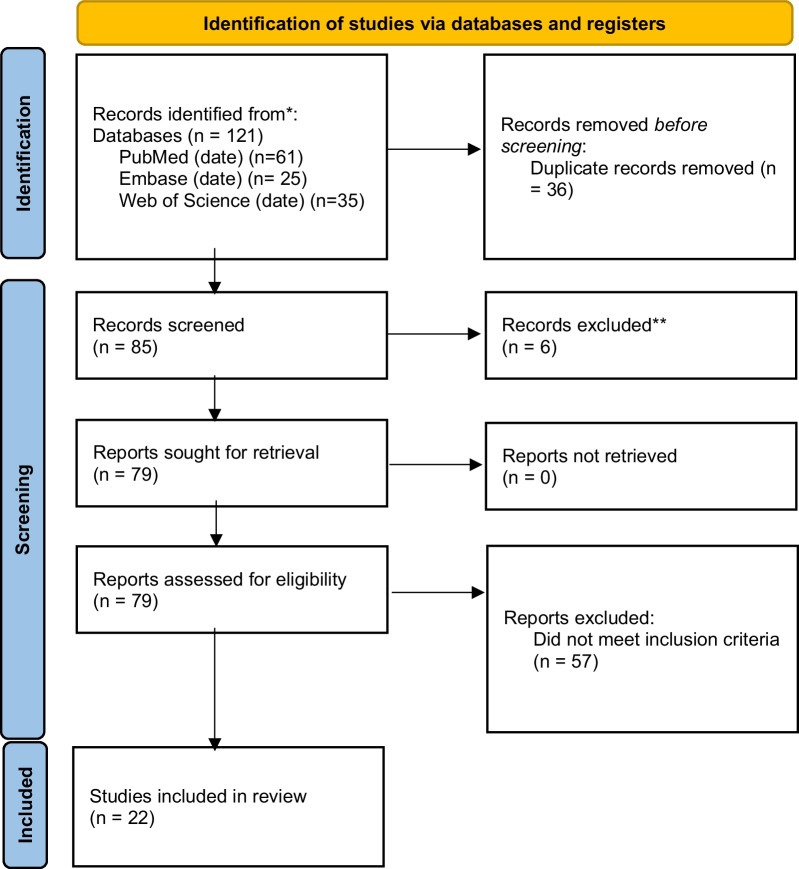
PRISMA flow diagram

The initial search yielded 85 unique records. The initial article search resulted in many studies in the 1980s/1990s, and some in more recent (within the past 10 years). By going back to 1980, we cast a very broad net but ensured a comprehensive search. There averaged 1–2 publications a year over the last 30 years, mostly in the mid-2000's and then 2020's. During the title and abstract screening 6 records were excluded, leaving 79 records for a full screening review. After applying the full inclusion and exclusion criteria, 57 records were excluded, mostly for not including the intervention of interest (“vending machines”) or population of interest (“people who use drugs”), leaving 22 studies in the study (see *Fig. **1* [19]).

### Characteristics of evidence: implementation constructs

Across the 22 articles, individual-level data were reported for an estimated total of 6,896 people who use drugs and 269 members of staff or community constituency groups. Data collection ranged from automated information gathered at the point of use of the machine to interviews and focus groups.

### Application of RE-AIM

Implementation constructs drawn from RE-AIM and used to characterize evidence across all articles include acceptability (or participant perspectives), reach, and real-world effectiveness.

### Acceptability (participant perspectives)

HRVMs were highly acceptable among PWUD and most staff of harm reduction programs. No study identified negative consequences, adverse events, or health issues associated with HRVMs. Concern expressed by staff and PWUD included an inherent lack of in-person services typically offered at harm reduction programs (e.g., counseling, linkage to care) [20, 21], poor lighting affecting safety [5], stigma for PWUD seen using the machine [22], and vandalism of the machine [20]. In one study, constituencies reported barriers as current political climate, lack of community level preparation, and cultural context [23]. HRVM are not associated with increases in crime rates, nor has loitering or related activity been observed [24] (Table 1).

**Table 1:** Summary of literature findings

Authorship	Year published	Study location	Sample size	Sample population	Reach evaluated	Effectiveness outcomes evaluated	Data collection method	Intervention evaluated	Underserved populations	CFIR domain
Cama, E., Brener, L., & Bryant, J	2014	New South Wales, Australia	183	PWUD	N/A	N/A	Anonymous surveys of fixed site, machine, and mobile syringe exchange users	Characteristics and attendance patterns of fixed site and HRVM users	Youth—no Race/ethnicity—no Rural/urban—no	Intervention characteristics Individual characteristics
Cossar, R., O’keefe, D., Dietze, P.M., & Jacka, D	2017	Melbourne, Australia	2795	PWUD	N/A	N/A	Demographic data collected along with time of use and supply distribution counts each time a HRVM user accessed the machine	HRVM use in Melbourne	Youth—yes Race/ethnicity—no Rural/urban—no	No domains identified
Cossar, R., O’keefe, D., Verdejo-Garcia, A., & Dietze, P.M	2017	Melbourne, Australia	74	PWUD	N/A	Individual-level syringe coverage (percentage of a PWID's injection episodes which are covered with a sterile syringe)	Structured survey of HRVM users, inclusive of the short-form UPPS-P Impulsive Behaviour scale	Association between individual-level syringe coverage, lack of premeditation, or positive urgency and HRVM use	None identified	Intervention characteristics Individual characteristics
Coupland, H., Henderson, C., Pritchard-Jones, J., Kao, S.C., Sheils, S., Nagy, R., O’Donnell, M., Haber, P. S., & Day, C. A	2022	Sydney, Australia	85	people who use drugs	N/A	N/A	Natural observation, survey and in-depth interview	HRVM use in Sydney	Youth—no Race/ethnicity—no Rural/urban—no	None
Day, C. A., White, B., & Haber, P. S. (2016)	2016	Australia	262	PWUD	N/A	N/A	SSP and HRVM user census, survey only of SSP participants	Addition of HRVM outside existing fixed site SSP	Youth—no Race/ethnicity—no Rural/urban—no	Intervention characteristics Individual characteristics
Deimel, D., Oswald, F., Carolin, B., & Stöver, H	2020	Germany	36	PWUD Program staff	N/A	N/A	Online questionnaire	HRVM that dispense syringes	Youth—no Race/ethnicity—no Rural/urban—yes	Inner setting Outer setting Intervention characteristics Individual characteristics
Dodding, J. & Gaughwin, M	1995	Australia	24	PWUD Program staff	N/A	N/A	Focus groups	N/A	Youth—yes Race/ethnicity—no Rural/urban—yes	Intervention characteristics Individual characteristics
Duplessy, C. & Reynaud, E. G	2014	Paris, France	N/A	N/A	N/A	N/A	Observations of syringe dispensing and collection trends via mechanical counting mechanisms on HRVM across Paris over a 12-year period	Expansion of existing vending machine program in Paris	None identified	Intervention characteristics Process
Islam, M. M., & Conigrave, K. M. (2007)	2007	Global	N/A	N/A	Yes—HRVMs reached high-risk, isolated, hidden populations of PWID who are otherwise not connected with SSPs	N/A	Literature review	N/A	Youth—no Race/ethnicity—no Rural/urban—no	Inner setting Outer setting Intervention characteristics Process
Islam, M. M., Conigrave, K. M., & Stern, T	2009	Sydney, Australia	94	Program staff	N/A	N/A	survey	Pilot study of 1 year implementation of vending machines	Youth—no Race/ethnicity—no Rural/urban—no	Outer setting Intervention characteristics
Islam, M. M., Stern, T., Conigrave, K. M., & Wodak, A	2008	NSW, Australia	167	PWID	Yes—access is increase by provision of service in evening hours	Injection risk behavior changes	Survey of HRVM users via face to face and machine-administered questionnaires	Existing machines in the community	Youth—yes Race/ethnicity—no Rural/urban—no	Inner setting Intervention characteristics Process
Islam, M. M., Wodak, A., & Conigrave, K. M	2008	Worldwide	N/A	N/A	Yes—HRVMs increase access to sterile injecting supplies, especially through added temporal availability	Injection risk behavior changes	Literature review	N/A	Youth—yes Race/ethnicity—no Rural/urban—no	Outer setting Intervention characteristics Individual characteristics
McDonald, D	2009	Canberra, Australia	180	PWID	Yes—existing fixed site users and new PWUD reluctant to access syringe service programs	N/A	Observation (administrative data), survey, program evaluation study	One-year pilot study implementation of vending machines	Youth—yes Race/ethnicity—no Rural/urban—no	Intervention characteristics Individual characteristics
Moatti, J. P., Vlahov, D., Feroni, I., Perrin, V., & Obadia. Y	2001	Marseille, France	343	PWUD	Yes—HRVM users are more likely to be younger and not enrolled in methadone programs	N/A	Self-administered questionnaire	Comparison of demographics and other traits of HRVM users vs. fixed site SSP participants	Youth—yes Race/ethnicity—no Rural/urban—no	Outer setting Intervention characteristics Individual characteristics
Obadia, Y., Feroni, I., Perrin, V., Vlahov, D., & Moatti, J. P	1999	Marseille, France	343	PWID	N/A	N/A	Survey /self-questionnaires	Evaluation of previous launch in 1996	Youth—yes Race/ethnicity—no Rural/urban—no	Intervention characteristics Individual characteristics Process
Otiashvili, D., Kirtadze, I., Vardanashvili, I., Tabatadze, M., & Ober, A. J	2019	Georgia	149	PWID	N/A	N/A	Interviews	N/A	Youth—no Race/ethnicity—no Rural/urban—no	Outer setting Intervention characteristics
Otiashvili, D., Irma Kirtadze, Tamar Mgebrishvili, Ada Beselia, Mzia Tabatadze, Irina Vardanashvili, & Ober, A.J	2022	Georgia	1,268	Program staff	Yes—the brand-new program reached 8% of the total population of PWID in the service region	Study defined effectiveness as "how well HRVM provided syringe access", including (1) the proportion of sterile syringes distributed via HRVM out of all syringes distributed via all types of needle and syringe programs, and (2) a difference in a number of syringes received by clients who used and who did not use HRVM	Stepped-wedge design, self-administered staff surveys, focus group of staff, focus groups of participants	Five sites at HIV prevention locations across Georgia	Youth—yes Race/ethnicity—no Rural/urban—no	Inner setting Outer setting Intervention characteristics Individual characteristics Process
Otiashvili, D., Kirtadze, I., Mgebrishvili, T., Beselia, A., Tabatadze, M., Otiashvili, N., Ober, A. J., & Iguchi, M. Y	2021	Georgia	800	PWID	N/A	N/A	Formative process, surveys, focus groups	Five sites at HIV prevention locations across Georgia	Youth—no Race/ethnicity—no Rural/urban—no	Intervention characteristics Process
Philbin, M. M., Mantsios, A., Lozada, R., Case, P., Pollini, R. A., Alvelais, J., Latkin, C. A., Magis-Rodriguez, C., & Strathdee, S. A	2009	Tijuana, Mexico	49	Stakeholders	N/A	N/A	Interviews and legal review	N/A	Youth—no Race/ethnicity—no Rural/urban—no	Outer setting Individual characteristics
Stark, K., Leicht, A., & Muller, R	1994	Berlin, Germany	313	PWID	N/A	N/A	Researcher-administered survey	Existing machines in the community	Youth—no Race/ethnicity—no Rural/urban—no	Inner setting Outer setting Intervention characteristics Individual characteristics Process
Uthurralt, N., McGlinn, A., O’Donnell, M., Haber, P. S., & Day, C. A	2022	Australia	N/A	N/A	N/A	N/A	Descriptive/observational, from administrative data	Expansion of hours/availability of HRVM and resulting impact on uptake of primary healthcare services at an existing fixed site SSP	Youth—no Race/ethnicity—no Rural/urban—no	No domains identified
White, B., Haber, P. S., & Day, C. A	2016	Sydney, Australia	153	Stakeholders	N/A	N/A	Researcher-administered survey	N/A	Youth—no Race/ethnicity—no Rural/urban—no	No domains identified

### Reach

Articles that included reach suggest that HRVM have potential use among diverse populations, but the evidence to support this is sparse. PWUD accessing vending machines were more likely to be younger [5, 6, 14, 20, 21, 25] and have a shorter drug use history [6, 22, 25, 26] than those who accessed services in other ways. There was evidence that vending machines engaged hidden or “harder to reach” populations [21] who use drugs less frequently [20] and are less connected to social services [25]. There was inconclusive evidence on any differences in utilization frequency by gender [5, 22], and in risk profiles (higher risk vs. less likely to engage in HIV risk behaviors) [6]. Lack of evidence can be explained by the simplistic technology of early HRVM that predated online data tracking systems.

None of the studies reported on race or ethnicity, impeding retroactive application of a racial equity lens. While some studies mentioned the collection of demographics in surveys or interviews, they do not incorporate them into analyses in a way that would identify the role of HRVM in addressing race disparities in syringe access and associated health outcomes. One review article included a reference to the role of race in Ireland in which hard to reach populations were listed (i.e., those with chaotic drug use patterns, mobile/homeless, and from an “ethnic minority” [21].)

HRVM users differed in a number of ways from participants who preferred fixed site SSPs. Women and young people were more likely to use HRVM than men and older people which is consistent with other study findings that identified the most common users of HRVM to be young and relatively new to injecting [27]. Moatti et al. [28] also found that people who preferred HRVMs tended to be younger, HIV-positive, and less likely enrolled in a methadone program. Notably, people preferring HRVMs also reported being less financially stable, and less likely to inject heroin as compared to people who preferred fixed site SSPs [24, 28]. Most people who access HRVM do so frequently, almost daily, and rely on HRVM as a primary source of supplies [5].

### Effectiveness

Among studies that examined effectiveness, the outcomes were mixed between clear effectiveness and inconclusive results, and largely focused on health outcomes, access and uptake of syringe services [20, 22, 23, 26]. Most cited implementation outcomes such as expanded after-hour availability for services [5, 14, 20–22, 25], greater anonymity [5, 20, 22], decreased syringe sharing [5, 6], increased access to supplies [14, 22], free products [22], and disease prevention [23]. The gap widens in regards to the impact of increased access to supplies other than syringes, because almost all articles focused on HRVM use for syringe distribution. For studies that did measure effectiveness, HRVM were most impactful for reducing syringe sharing [5, 6], increasing access to services [14], and serving “hard to reach” PWID [21]. One article evaluated the association between the implementation of naloxone dispensation at HRVMs and overdose fatalities; their results suggested that significant reductions in opioid-involved overdose fatalities occurred in the year following naloxone dispensation at HRVMs [29].

### Application of CFIR

Despite articles spanning multiple decades, countries, and populations served, facilitators and barriers within all 5 CFIR Domains were observed in the 22 articles included in this study. In the sections below, we discuss the constructs that rose to the surface within each domain. Not all articles addressed implementation or CFIR, and there are many more CFIR constructs than are represented below.

### Intervention characteristics

#### Facilitators of harm reduction vending machines

The CFIR constructs within the intervention characteristic domain that facilitate implementation of HRVMs include relative advantage, location and cost, adaptability, and design, quality, and packaging. The most prevalent intervention characteristic is HRVM’s *relative advantage* when compared to other geographically located syringe or naloxone access points. HRVM regularly attract participants who are not accessing fixed site locations or pharmacies [30]. Further, HRVM address barriers with brick and mortar locations including but not limited to 24/7 access [20]. All-hours access is a critical facilitator, and many HRVM transactions occur during non-working and holiday hours [27]. However, while there is an advantage at times for HRVMs as compared to SSPs, multiple studies note that HRVMs function best as a complementary service to storefront or mobile exchange and a backup service for SSP clients after hours [5, 6, 20, 21, 28].

Other subdomains include location and cost. A major implementation factor to consider is the importance of location selection: to maximize client privacy, convenience, anonymity, and reduce travel time [5, 14, 20, 21]. Design quality and packaging also emerged as important facilitators of the “normalizing” of the machines [31]. Anonymity, easy transportation access, and co-location in an area of high drug activity did not seem to be advantageous *enough* to motivate users who lived *outside* of the neighborhood to access the HRVM [24]. When other alternative modes of access are lower-barrier, such as home or mail delivery, PWUD may prefer those modes over HRVMs [27]. This connects to the outer setting construct of patient needs and resources and community networks. Specifically, an organization needs to be aware of the needs and networks of PWUD to best place and stock a HRVM.

Free cost to people who use HRVMs was a motivator to access HRVM in France, where 62.6% of people surveyed reported that access to free syringes was a main reason for seeking out the HRVM [28]. If free is not feasible, McDonald [5] did show that a very minor cost (less than AUD 2.00) was acceptable to participants.

A few studies emphasized *adaptability* of the intervention [14, 26, 31]. Adaptability relies on a definition of the "core components" (the essential and indispensable elements of the intervention itself) versus the "adaptable periphery" (adaptable elements, structures, and systems related to the intervention and organization into which it is being implemented) of the intervention. This construct connects both to the organization’s ability/willingness to adapt to the needs of participants, and the need for the resources to do so. The intervention is able to be adapted to include new supplies and more frequent refills based on participant feedback about their needs. For example, the HRVMs in the nation of Georgia incorporated the needs of local populations by providing supplies to both the general population and PWID in the same machine to avoid stigmatization [31].

#### Barriers to harm reduction vending machines

The two identified CFIR constructs that act as barriers to implementation include lack of quality of evidence and design. HRVMs constituencies’ perceptions of the quality and validity of evidence can be a barrier to decision-making around both HRVM initiation and implementation [16]. Some studies have documented assumed or anticipated adverse effects of HRVMs among constituencies, including increasing drug use [23], syringe litter [32], or increasing syringe use among young people [20, 32]. However, it should be noted that these assumptions were refuted in others [24, 33], and the motivation of this scoping review is to summarize an increased quality of evidence.

Design emerged as a potential facilitator to implementation as discussed above, but the proper functioning of the machine is a barrier. The reliability and operability of the HRVM is critical to success, emphasized by both people who use HRVMs and broader community constituencies. It is important to ensure that HRVMs are well stocked and remain stocked. It is also important to ensure the “mechanical reliability” and “durability of the machine” [14]. Incidents of malfunction include electrical, software and mechanical problems, could be enough to prevent return visits from PWUD. Additionally, at least one study mentioned vandalism as a potential barrier to continued use, particularly over time [5].

### Outer setting

#### Facilitators of harm reduction vending machines

The most prevalent outer setting construct is patient needs and resources, defined as the extent to which patient needs, and the barriers and facilitators to meet those needs, are known and prioritized by the organization. This expectation builds on the identified inner setting construct of organizational climate; it's critical, and complimentary, for organizations to be willing to adapt the intervention in response to participant feedback. Participants can, and should, be consulted on the location and contents of the HRVM to ensure it meets their needs. Moreover, a connection with drug user networks is helpful to inform the location of the machine was critical to success tying needs and resources to networks and communication [21]. Studied HRVM met participant needs. In areas where programs offered only single syringe dispensing, HRVM met a need for greater quantities of syringes [33]. HRVM users received more syringes than their counterparts who used fixed site SSPs, a statistically significant difference [27]. Many articles cited a need from staff and participants for a greater variety of harm reduction supplies, a clear benefit from efforts to obtain ongoing feedback about the intervention [33].

Finally, cosmopolitanism emerged as a strategy to mitigate concerns about HRVM and encourage people to go to needed in-person services or connect with program staff during a crisis. Over time, an Australian program observed that HRVM users began to access the staffed services. Staff believed their connection with other service providers helped them better meet the needs of their program participants [34].

#### Barriers to harm reduction vending machines

External policy and incentives, or politics, were generally referred to as a benefit and a barrier. Many organizations trying to implement HRVM were met with positive responses from external partners [33]. For example, community attitudes and vandalism risk were reduced when local government bodies were involved in HRVM implementation [14]. In contrast, lack of cultural support and disinterest from the government were seen as challenges to implementation in Tijuana [23].

Also, some PWID did not want to use HRVMs because of fear of police or lack of anonymity associated with accessing services in a public space [27]. Police are a critical concern for HRVM use in many communities, particularly Black and Brown communities in the US that have been historically over-policed and over-criminalized for drug use.

### Inner setting

#### Facilitators of harm reduction vending machines

Inner setting constructs were the least prevalent across all studies. In those that identified them, implementation climate and available resources emerged as important facilitators of HRVM implementation. The climate was such that organizations were willing to make changes based on feedback from participants, which contributed to satisfaction with the HRVM [26]. An appropriate climate is one of acceptance, enthusiasm, commitment, and support for people who use drugs. Without these elements, organizations will have a more difficult time connecting with and engaging participants in feedback on HRVM operations.

Cost is another notable facilitator and concern for HRVM operators and related to the need for external policy and incentives, government support, and inner setting resources. Programs need to be able to spend money to ensure the machines are full and reliable [20]. Finally, studies emphasized the cost effectiveness of HRVM, particularly when compared to the expense of staffing a 24 h syringe service or other program fixed site location [14, 21].

#### Barriers to harm reduction vending machines

Cultural barriers at other access points, such as being male-dominated or judgmental, contributed to people being more likely to use HRVM [21, 25]. This speaks to an inner setting of acceptance of people who use drugs, non-stigmatizing communication, and support of participants. This was most notable among women and younger people, who were more likely to convert from using only fixed site services to also using HRVMs than were men and older people, because of perceived and experienced stigmatization [25, 27].

### Characteristics of individuals

#### Facilitators of harm reduction vending machines

Knowledge and beliefs of the intervention among staff emerged as a facilitator. HRVM implementers had to be knowledgeable about them in order to effectively address community concerns and proactively promote the intervention in new spaces. This includes familiarity with the published literature and other evidence backing the intervention. Organizations should be prepared for continuous public relations efforts and staff should cultivate savviness with communications [33]. No barriers emerged, except as the inverse of the identified facilitators.

### Process

#### Facilitators of harm reduction vending machines

The most prevalent construct within the process domain is engagement. Multiple articles referenced engagement with PWUD to understand needed supplies, barriers to access, and ideal location for vending machine placement [26]. The organization should expect to change aspects of the HRVM based on formally collected participant feedback [26] and continuously distribute information on the location of placed machines [27].

Planning and evaluation was also important to the successful implementation of HRVM. Formative research developed the program structure prior to and during implementation. This includes assessing needs, preferences, barriers, and facilitators to implementation [31]. Multiple articles described how HRVM adapted the intervention based on participant feedback collected during evaluation. Implementors thoughtfully considered data collection at the point of use of HRVMs, particularly as the technology advanced, in order to best capture intervention performance and reach through routine data collection [21, 27].

#### Barriers to harm reduction vending machines

The inverse of facilitators, or a lack of planning, evaluation and engagement, can lead to implementation barriers. If PWUD are not engaged, they may not know where HRVMs are located or maintain concerns about police intervention [6]. Educating governing bodies could help with preventing vandalism and addressing community pushback. Finally, political leaders and local decision makers may influence an HRVM’s ability to be placed in a certain location [27].

## Limitations

Authors identified two types of limitations: existing article limitations and reviewer bias. All of the studies that informed this scoping review had limitations. Primarily, most articles were qualitative and relied on small sample sizes. No randomized clinical trials were found, an expected yet noteworthy limitation, in that it suggests a need for rigorous evaluation of vending machines. As are most studies with PWUD, samples were convenient or relied on those who were already engaged with an SSP. This sampling design can miss people who are even more marginalized and not connected to any harm reduction services. Moreover, summarizing literature across a broad time span limits the distinct cultural, technological, environmental, legal, and other differences that might impact PWUD beliefs and behavior over time.

Another limitation of the existing literature is a sole focus on syringes and people who inject drugs that are too narrow to apply to a contemporary US context. Health outcomes measured are limited to prevention of infectious disease, HIV, or change in immediate behaviors, like reductions in syringe sharing. There is currently only one study about HRVMs that considers overdose-related outcomes. People who inhale, smoke, or snort drugs are also at risk for HIV, HCV, and overdose and often not included in samples recruited at SSPs. This is particularly pertinent since the integration of fentanyl in the heroin supply in the US has drastically increased overdose deaths among users of all types of substances. For contemporary implementation and evaluation of HRVM, SSP providers, evaluators, and harm reduction researchers must go beyond the syringe and consider multiple types of paraphernalia to engage a broader community of people using drugs.

In addition, the authors recognize the limitations of using the CFIR framework to identify facilitators and barriers. There was a small proportion of discussion of barriers to implementation compared to facilitators. There is potential for author bias toward a positive view of HRVM and their implementation, with which more facilitators than barriers would be identified in the literature. The general lack of implementation science articles also contributes to this limitation.

## Recommendations

Relatively rapid development of community-based harm reduction vending machines in the US would benefit from the experience of over 30 years of implementation abroad. Contemporary HRVM should consider maximizing availability as close to 24/7 as possible, to complement other harm reduction program hours, offering syringe disposal options, collecting data, and ensuring anonymity of their use. The available literature on HRVM reveals strong potential for addressing health disparities when thoughtfully implemented.

From the planning stages, HRVM implementers should identify methods for establishing strong feedback loops between them and their program participants. This feedback is critical for identifying the best location, design and packaging of supplies, and addressing issues throughout implementation and sustainment. The readiness and planning stage will most benefit implementation if it also includes constituency engagement, particularly on the quality and validity of the evidence of HRVM. Readiness assessments can ask program participants about placement of the machine, the supplies that should be stocked, travel preferences, willingness to pay for supplies, and about any anticipated barriers to access. Throughout implementation, it's critical that programs continue to gather feedback from participants and adapt as necessary. This is particularly important in contemporary US context where SSPs continue to get push back from communities primarily due to NIMBYism.

Rigorous study design, diverse sampling methods and the incorporation of health outcomes data would improve future studies. Given high levels of acceptability of HRVM, future studies can prioritize other aspects of the CFIR, RE-AIM or other implementation science frameworks. Finally, this review touched upon multiple CFIR and REAIM constructs and invites further study into the ability of HRVM to reach people of racial minorities.

## Data Availability

All data used in this manuscript were created by the authors and are represented in Table 1, *summary of literature findings.*

## Footnotes

1 In this review, people who use drugs (PWUD) and people who inject drugs (PWID) are used interchangeably. PWID is applied when it is the specific population of a cited study, or the term used in a cited article. Studies do not differentiate between use of opioids, stimulants, or other drugs, and most provided injection supplies.
